# Role for Retinoic Acid-Related Orphan Receptor Alpha (RORα) Expressing Macrophages in Diet-Induced Obesity

**DOI:** 10.3389/fimmu.2020.01966

**Published:** 2020-08-27

**Authors:** Emily Hams, Joseph Roberts, Rachel Bermingham, Andrew E. Hogan, Donal O'Shea, Luke O'Neill, Padraic G. Fallon

**Affiliations:** ^1^School of Medicine, Trinity Biomedical Sciences Institute, Trinity College Dublin, Dublin, Ireland; ^2^Department of Biology, National University of Ireland, Maynooth, Ireland; ^3^Obesity Immunology Research, St. Vincent's University Hospital and University College Dublin, Dublin, Ireland; ^4^School of Biochemistry and Immunology, Trinity Biomedical Sciences Institute, Trinity College Dublin, Dublin, Ireland

**Keywords:** RORα, macrophage, inflammation, metabolism, obesity

## Abstract

The transcription factor RORα plays an important role in regulating circadian rhythm, inflammation, metabolism, and cellular development. Herein we show a role for RORα-expressing macrophages in the adipose tissue in altering the metabolic state of mice on a high-fat diet. The expression of *Rora* and *RORA* is elevated in white adipose tissue from obese mice and humans when compared to lean counterparts. When fed a high-fat diet *Rora* reporter mice revealed increased expression of *Rora*-YFP in macrophages in white adipose tissue deposits. To further define the potential role for *Rora*-expressing macrophages in the generation of an aberrant metabolic state *Rora*^fl/fl^LysM^Cre/+^ mice, which do not express *Rora* in myeloid cells, were maintained on a high-fat diet, and metabolic parameters assessed. These mice had significantly impaired weight gain and improved metabolic parameters in comparison to *Rora*^fl/fl^ control mice. Further analysis of the immune cell populations within white adipose tissue deposits demonstrates a decrease in inflammatory adipose tissue macrophages (ATM). In obese reporter mouse there was increased in *Rora*-YFP expressing ATM in adipose tissue. Analysis of peritoneal macrophage populations demonstrates that within the peritoneal cavity *Rora*-expression is limited to myeloid-derived macrophages, suggesting a novel role for RORα in macrophage development and activation, which can impact on metabolism, and inflammation.

## Introduction

The transcription factor retinoic acid receptor-related orphan receptor alpha (RORα) is a member of the nuclear hormone receptor superfamily, which provides a bridge between hormonal, nutritional, and pathophysiological signaling and gene regulation. RORα itself has been identified as having roles in neural development, metabolism, cellular differentiation, immune regulation, and circadian rhythm. Indeed, staggerer mice (*Rora*^sg/sg^), which express a truncated form of the RORα protein due to a spontaneous mutation in the *Rora* gene, show aberrant immune responses (1–4).

Studies on RORα in the context of immunity have focused primarily on two aspects; regulation of immune signaling pathways, and involvement in immune cell development. Expression of RORα is induced in tissue and cells, including macrophages, in response to inflammatory stimuli, suggesting a role in the immune response (5, 6). Indeed, RORα has been implicated in the inflammatory response to the TLR agonist lipopolysaccharide (LPS) through its ability to down-modulate NF-κB signaling and impair activation of the NLRP3 inflammasome (1, 2, 5).

RORα has also been linked with allergic and autoimmune diseases, with *RORA* implicated in the development of asthma (7) and also increased susceptibility to multiple sclerosis (8). Similarly, in experimental models *Rora*^sg/sg^ mice show attenuated allergic inflammatory responses in allergen-induced lung inflammation (9); while mice deficient in RORα and RORγ are completely refractory to the development of experimental autoimmune encephalomyelitis (10). Furthermore, in the skin RORα-expressing T regulatory cells are involved in allergic inflammation (11). In addition to the inhibition of inflammatory signaling pathways, RORα has also been identified as critical in the development of Th17 cells and group 2 innate lymphoid cells (ILC2), which may explain the attenuation of allergic and autoimmune conditions seen in these studies (10, 12).

Within the context of metabolism RORα has an important role in lipid and glucose metabolism. *Rora*^sg/sg^ mice, which are resistant to diet-induced obesity, show increased insulin sensitivity with increase adipocyte lipid storage capacity without harboring hyperlipidemia or hepatic steatosis. This increase in energy storage in *Rora*^sg/sg^ mice is believed to be mitigated in part through increased “browning” of white adipose tissue (WAT) in these mice. During this process, increased energy expenditure occurs as a result of heat generation associated with increased expression of uncoupling protein 1 (UCP1). In *Rora*^sg/sg^ mice there is increased UCP1 in both inguinal BAT and WAT (13).

In this study, we demonstrate a role for the *Rora* expressing macrophages in obesity. *Rora*-YFP reporter mouse were used in a diet-induced model of obesity to identify Rora expressing cells within the adipose tissue. The primary cell populations expressing *Rora*-YFP in adipose tissue were inflammatory adipose tissue macrophages (ATM), with increased frequency of ATM in obese animals. When fed a high fat diet *Rora*^fl/fl^LysM^Cre/+^ mice, which have a myeloid-cell specific deletion of *Rora*, developed increased weight gain with glucose sensitivity compared to control animals. We further show that deletion of *Rora* in myeloid cells is sufficient to impact on genes associated with thermogenesis, suggesting *Rora*-expressing macrophages not only impact on inflammation and obesity, but also regulate metabolic gene expression within the adipose tissue. This study highlights the importance of *Rora*-expressing macrophages in the context of the inflammation and metabolic alterations that underlie obesity.

## Materials and Methods

### Animals

C57BL/6J (wild type; WT), *Rora*^*s*^^g/sg^ (JAX Strain: 002651), R26R-EYFP (JAX Strain: 006148), *Lyz2*^tm1(cre)Ifo^ (JAX Strain 004781) were purchased from Jackson Laboratories (Bar Harbor, MD, USA). Conditional *Rora* floxed mice were generated (Lexicon Pharmaceuticals, USA) and homozygous mice crossed to *Lyz2*^tm1(cre)Ifo^ (referred to herein as LysM^Cre/+^) to generate animals with a conditional deletion of *Rora* in cells of a myeloid lineage, and IL-7Ra^Cre/+^ (14) for conditional deletion of *Rora* in cells expressing IL-7Rα (*Rora*^fl/fl^*Il7r*^Cre^ mice). *Rora*^tm1(cre)Ddmo^ mice (15) were crossed with R26R-EYFP mice to generate *Rora*-YFP reporter mice (referred to herein as *Rora*-YFP). Male mice were used in all studies. Animals were housed in a specific pathogen-free facility in individually ventilated and filtered cages under positive pressure. All animal experiments were performed in compliance with the Health Product's Regulatory Authority and approved by the Trinity College Dublin's BioResources ethical review board.

It is relevant that due to the importance of RORα in regulating circadian rhythm (16), and the corresponding circadian rhythm known to govern circulating monocytes and thus potentially impacting upon the inflammatory response (17), all experiments on mice and tissue isolations were performed at 10–12 a.m. to ensure no variations occurred due to alterations in the circadian rhythm due to time differences in experiments.

### Human Adipose Tissue Samples

RNA was isolated from snap-frozen omental adipose tissue biopsies from obese patients (body mass index, BMI < 50) undergoing elective bariatric surgery and from control patients with a healthy BMI (18–23) undergoing elective abdominal surgery. Clinical studies were approved by the St. Vincent's University Hospital, Dublin, Ethics committee. Written informed consent was obtained from each patient before commencement of research activities.

### High Fat Diet and *in vivo* Metabolic Testing

A mouse model of diet-induced obesity by feeding high fat diet (HFD) was as described (24, 25). Age-matched male *Rora*^fl/fl^ and *Rora*^fl/fl^LysM^Cre/+^ mice were fed a HFD (60% kcal fat; D12492; Research Diets, Inc., NJ, USA) or control diet (10% kcal fat; D12450J) *ad libitum* for 16 weeks. Male mice were used for all HFD studies due to the published differences in the development of metabolic syndrome between males and females, with male mice on a C57BL/6 mice showing a higher predisposition to weight gain and metabolic dysfunction than female counterparts (18, 19). Glucose tolerance was assessed in mice fasted overnight and challenged with 2 g/kg glucose i.p. Insulin tolerance was tested in mice fasted for 4 h and challenged with 0.75 mU/g human insulin i.p. Blood glucose was measured prior to injection and at 30, 60, and 120 min post-injection using a glucometer (Abbott Laboratories, IL, USA). All metabolic analyses were undertaken between 10.00 and 12.00, all weight measurements were taken at 10.00.

### Serum Transaminase Assay

Free triglycerides and alanine transaminase (ALT) and aspartate transaminase (AST) were measured in the serum of *Rora*^fl/fl^ and *Rora*^fl/fl^LysM^Cre/+^ mice after 16 weeks on HFD. Serum was assessed for triglyceride levels using the Abnova triglyceride quantification kit (Heidelberg, Germany) following the manufacturer's instructions. ALT and AST activity was determined in the serum using commercial kits from Abcam (Cambridge, UK) following the manufacturer's instructions.

### *In vivo* Antibody Treatment

Groups of age-matched male *Rora*^Cre^RosaYFP mice were treated, using a method previously described (20), with IL-4/anti-IL-4 monoclonal antibody (mAb) complexes (IL-4c), thioglycollate (4% w/v; Sigma, Wicklow, Ireland), or thioglycollate + IL-4c i.p. IL-4c was prepared by incubating 5 μg recombinant mouse IL-4 (Peprotech, London, UK) with 25 μg anti-lL-4 mAb (clone 11B11, BioXcell, NH, USA). Briefly mice were injected with PBS or IL-4c i.p. on days 0 and 2. Mice were also treated with thioglycollate (day 0) or thioglycollate (day 0) plus IL-4c (day 0 and 2). Peritoneal cells were collected from all mice on day 4 by lavaging the peritoneal cavity with ice-cold PBS for flow cytometry.

### Adipose Tissue Stromal Vascular Cell Isolation

The stromal vascular fraction was isolated from adipose tissue and prepared for flow cytometry or culture. Briefly, the adipose tissue deposits (epididymal and inguinal white adipose tissue; E-WAT and I-WAT, respectively) were removed, weighed, minced and digested with 1 mg/ml Collagenase D from *Clostridium histolyticum* (Roche, Dublin, Ireland) for 30 min at 37°C for 30 min with shaking. Digested tissue was passed through a 70μM cell strainer and cells pelleted at 1,500 rpm for 5 min. Red blood cell contamination was removed by incubation with a hypotonic lysis solution. Cells were used for flow cytometry or cultured for 2 h in RPMI-1640 supplemented with 2 mM L-glutamine, 100 U/ml penicillin and 100 μg/ml streptomycin at a density of 2 × 10^6^ cells/ml at 37°C with 5% CO_2_. Gene expression was assessed in adherent cells.

### Murine Macrophage Isolation and Culture

Peritoneal macrophages were cultured from naive C57BL/6J, *Rora*^sg/sg^ and *Rora*^Cre^Rosa-YFP mice as previously described (21). Briefly, the peritoneal cavity was lavaged with 5 ml ice-cold PBS and the resulting cells plated at 2 × 10^6^ cells/ml in RPMI-1640 supplemented with 2 mM L-glutamine, 100 U/ml penicillin and 100 μg/ml streptomycin and incubated for 2 h at 37°C with 5% CO_2_, and any non-adherent cells removed. Adherent cells were collected for gene expression analysis.

Bone marrow derived macrophages (BMDM) were cultured from the tibia and fibula of mice as previously described (21). Briefly, cells were flushed from the tibia and fibula to prepare a single cell suspension. After red blood cell lysis, the resultant cells were cultured at 3 x 10^6^ cells/ml in RPMI-1640 supplemented with 2 mM L-glutamine, 100 U/ml penicillin, 100 μg/ml streptomycin and 10% fetal calf serum, with 20% L929 supernatant at 37°C with 5% CO_2_ for 7 days. BMDM were collected for gene expression analysis after 7 days culture. Separately, BMDM were stimulated with IL-4 (20 ng/ml; Peprotech, London, UK) or ultra-pure LPS (100 ng/ml; from *Escherichia coli* 0111:B4 strain, InvivoGen, France) for 48 h and cells collected for flow cytometry.

### Flow Cytometry

Surface marker expression was assessed by flow cytometry with data collection on a CyAn ADP (Beckman Coulter, High Wycombe, UK) and data analyzed using FlowJo software (Tree Star, OR, USA). Cells, isolated as previously described, were stained with BD Biosciences (Oxford, UK) mAbs; Siglec-F-PE (E50-2440), CD45.2-PE-CF594 (104), CD11b-APCCy7 (M1/70); eBioscience (Loughborough, UK) mAb; MHC class II-eFluor 450 (MS/114.15.2); and BioLegend (London, UK) mAbs; F4/80-FITC (BM8), Ly6C-PerCP (HK1.4), CD64-APC (X54-5/7.1), CD9-PECy7 (MZ3), CD206-PECy7 (C068C2), and CD115-PE (CSF-1R). Prior to surface staining, cells were incubated with LIVE/DEAD Fixable Aqua stain (Molecular Probes, Invitrogen, Dublin, Ireland) to isolate dead cells. Using appropriate controls, quadrants were drawn and data were plotted on logarithmic scale density-plots.

### RNA Isolation and Real-Time PCR

RNA was isolated from cultured cells and snap frozen adipose tissue deposits. For all cultured cells, RNA was isolated using the RNeasy kit (Qiagen, Hilden, Germany); RNA was isolated from adipose tissue samples using phenol/chloroform extraction. Briefly ~50 mg tissue was homogenized in 500 μl TRIzol® (ThermoFisher, Loughborough, UK), incubated on ice for 5 min and cellular debris removed by centrifugation. Chloroform (1:5 v/v) was added to the TRIzol suspension, mixed by vortex and incubated for 3 min, then separated by centrifugation. The aqueous layer was collected and RNA precipitated using 1:1 (v/v) isopropanol. RNA was pelleted by centrifugation and reconstituted in DEPC treated H_2_O. Resultant RNA was reverse transcribed using the Quantitect reverse transcription kit incorporating a genomic DNA elimination step (Qiagen, Hilden, Germany).

Real-time quantitative PCR was performed on an Applied Biosystems StepOne Plus sequence detection system (Applied Biosystems, Dublin, Ireland) using pre-designed TaqMan gene expression assays specific for murine *Rora* (Mm00443103_m1), *Nos2* (Mm00440485_m1), *Arg1* (Mm00475988_m1), *Ccl2* (Mm00441242_m1), *Tnf* (Mm00443258_m1), *Chil3* (Mm00657889_mH), *Ucp1* (Mm01244861_m1), *Elovl3* (Mm00468164_m1), *Cidea* (Mm00432554_m1), *Cpt1b* (Mm00487191_g1), *Ppargc1* (Mm01208835_m1), and human *RORA* (Hs00536545_m1) and normalized to 18S.

### Statistics

Statistical analysis was performed using GraphPad 8. Results are presented as mean ± SEM. Statistical difference between groups were analyzed by unpaired Student's *t*-test with Welch correction or ANOVA and Tukey's multiple comparison test. *P*-values were considered significant when *P* > 0.05.

## Results

### *Rora* Expression Is Increased in Adipose Tissue Macrophages in Obesity

**Figure 1:**
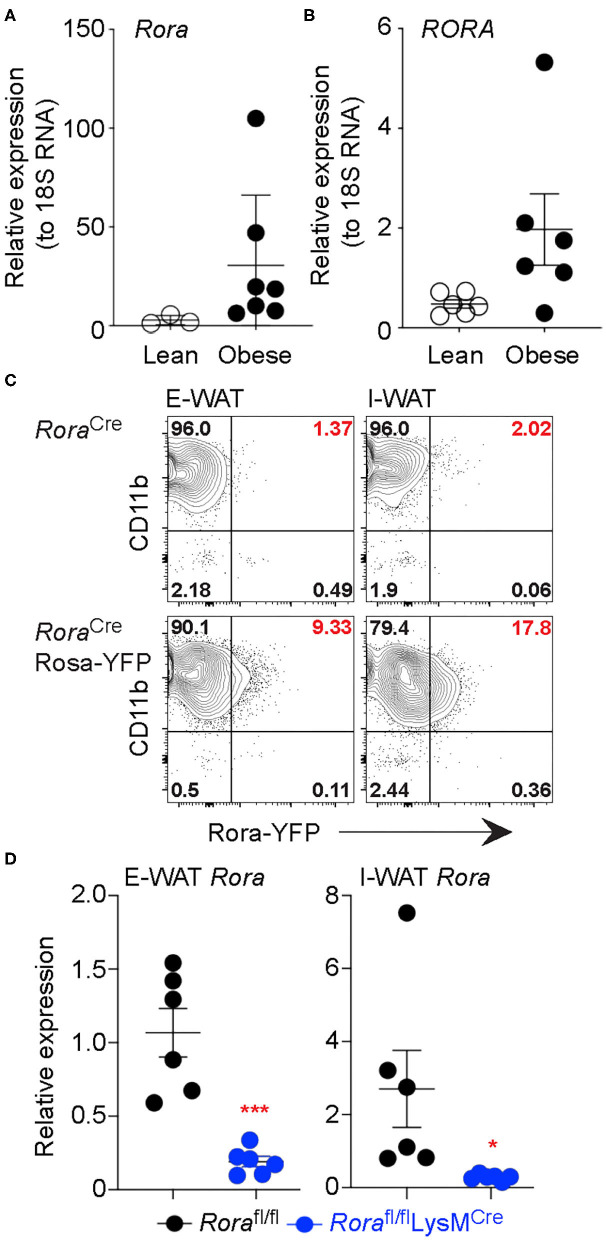
*Rora* expression is increased in myeloid cells in adipose tissue isolated from obese subjects. Expression of the gene encoding RORα was determined in mice (*Rora*) and human (*RORA*) (**A,B**, respectively). RNA was isolated from the E-WAT isolated from both lean and obese humans and mice, gene expression was quantified by qPCR and normalized based on 18S expression (*n* = 3 lean mice, *n* = 7 obese mice; *n* = 6 lean or obese patients). *Rora*^Cre^ and *Rora*^Cre^Rosa-YFP mice were fed a high-fat diet for 12 weeks and flow cytometry performed on epididymal and inguinal white adipose tissue (E-WAT and I-WAT, respectively). Cells were gated as Live/dead^−^CD45^+^ and assessed for *Rora*-YFP expression (**C**, data is representative of 3 *Rora*^Cre^ and 7 *Rora*^Cre^Rosa-YFP mice). **(D)** *Rora*^fl/fl^ and *Rora*^fl/fl^LysM^Cre/+^ mice were fed a high-fat diet for 16 weeks and RNA isolated from E-WAT and I-WAT; expression of *Rora* was assessed relative to 18S (*n* = 6). Data is representative of mean ± SEM. Student's *t*-test: *P < *0.05*, ***P < *0.001*.

RORα has previously been associated with metabolic dysfunction, with key roles in lipid and glucose metabolism. Indeed, RORα-deficient staggerer mice are protected against age- and diet-induced obesity, hepatosteatosis and insulin resistance (3). The decreased adiposity in these mice is associated with decreased triglyceride deposition and reduced expression of a number of genes associated with lipid metabolism, including apolipoprotein A-1 (*apoA1*) and apolipoprotein C-III (*apoCIII*) (22, 23). Further analysis using a liver-specific conditional deletion of *Rora* (RORα^LKO^) demonstrated an increase in proliferators-activated receptor-γ (PPARγ), which resulted in impaired negative self-regulation thus protecting against hepatosteatosis (26). These studies clearly demonstrate a role for RORα in regulating the processes that underlie obesity. Indeed, we see increased expression of *Rora*/*RORA* gene expression in white adipose tissue isolated from obese subjects, both mouse and humans, respectively, when compared to their lean counterparts (Figures 1A,B).

Obesity is considered an inflammatory condition, mediated by the inflammatory cell milieu in the adipose tissue and heavily influenced by the ATM, with inflammatory macrophages associated with an obese state (27). To determine the cellular location of RORα we generated a *Rora* reporter mouse, by interbreeding a *Rora*^Cre^ mouse with R26R-YFP to generate a strain co-expressing *Rora* and YFP, enabling *Rora* expression to be visualized as YFP by flow cytometry. We confirmed detected YFP^+ve^ cells express *Rora* by sorting YFP^+ve^ and YFP^−ve^ CD45^+ve^ populations from naïve splenocytes and assessing *Rora* gene expression in the sorted populations by RT-PCR (Supplementary Figures 1A,B). We fed male 8 week-old *Rora*^Cre/+^ control and *Rora*-YFP mice a high-fat diet for 16 weeks and isolated the E-WAT and I-WAT for flow cytometry. In AT of obese mice CD11b^+^ cells were the dominant *Rora*-YFP expressing CD45-expressing cell population, with CD4+ T cells and type 2 innate lymphoid cells (ILC2), cell subsets associated with high expression of RORα, representing just 2 and 0.6% of *Rora*-expressing cells, respectively, in obese AT, CD45^−ve^ cells in the E-WAT did not express *Rora*-YFP (Figure 1C, Supplementary Figures 2A,B). Indeed, ILC2s are widely associated with a lean phenotype and decrease in number in obese adipose tissue, which suggests that RORα may play differential roles in regulating metabolic disease dependent upon the cell type in which it is expressed (24, 25). Interestingly expression was higher in the inguinal adipose tissue, supporting differential effects of RORα in E-WAT and I-WAT in the context of thermogenesis (13). Confirming the expression of *Rora* in adipose tissue myeloid cells, we show decreased *Rora* expression in AT isolated from *Rora*^fl/fl^LysM^Cre/+^ mice compared to *Rora*^fl/fl^ animals (Figure 1D).

### Myeloid Cell Expression of RORα Results in Exacerbated Metabolic Distress

**Figure 2:**
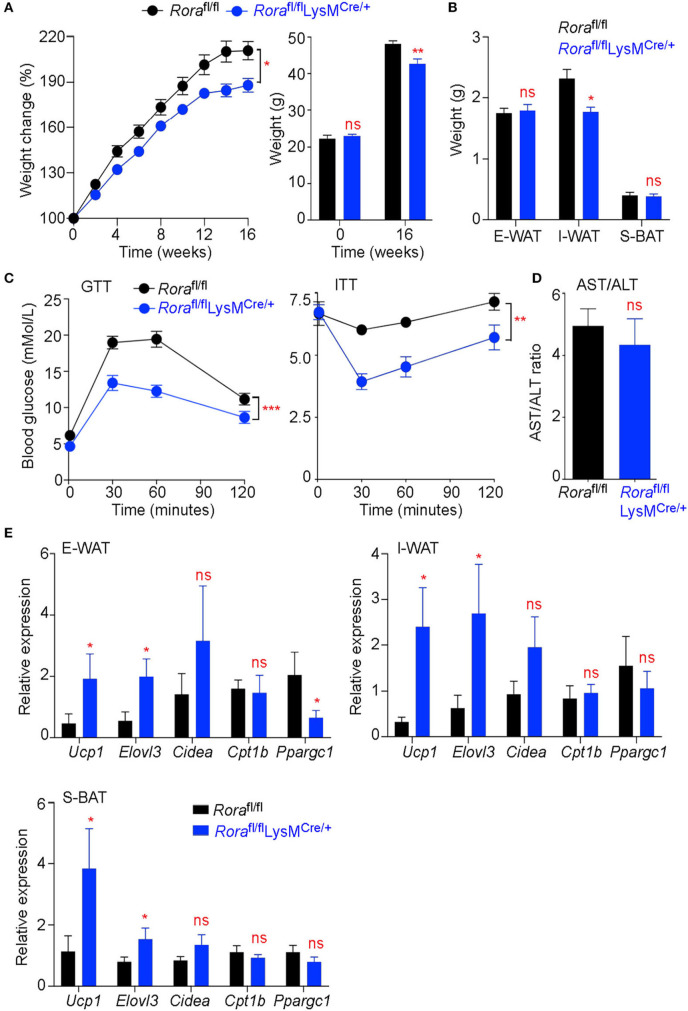
*Rora*^fl/fl^LysM^Cre/+^ mice show decreased weight gain and improved metabolic function associated with increased expression of genes associated with thermogenesis. Groups of *Rora*^fl/fl^ and *Rora*^fl/fl^LysM^Cre/+^ mice were fed a high-fat diet (HFD; 60% fat) for 16 weeks and weight monitored weekly, percentage weight gain was calculated from the starting weight of each animal and starting and final weight is quantified for each group (**A**, *n* = 11 *Rora*^fl/fl^; *n* = 19 *Rora*^fl/fl^LysM^Cre/+^). E-WAT, I-WAT and S-BAT (subcutaneous brown adipose tissue) weight was determined after 16 weeks on HFD (**B**, *n* = 7 *Rora*^fl/fl^; *n* = 6 *Rora*^fl/fl^LysM^Cre/+^). Glucose and insulin tolerance tests (GTT and ITT, respectively) were performed on mice after 14 and 15 weeks on HFD respectively (**C**, *n* = 8 *Rora*^fl/fl^; *n* = 6 *Rora*^fl/fl^LysM^Cre/+^). The serum AST/ALT ratio was calculated from AST and ALT activity tests on the serum from *Rora*^fl/fl^ and *Rora*^fl/fl^LysM^Cre/+^ mice after 16 weeks on HFD (**D**, *n* = 8 *Rora*^fl/fl^; *n* = 6 *Rora*^fl/fl^LysM^Cre/+^). E-WAT, I-WAT, and S-BAT were excised after 16 weeks on HFD (*n* = 6 *Rora*^fl/fl^; n = 6 *Rora*^fl/fl^LysM^Cre/+^). RNA was isolated and expression of genes associated with the metabolic and thermogenic function of the adipose tissue were assessed **(E)**. All data is representative of mean ± SEM. Student's *t* test: *P < *0.05*, **P < *0.01*, ***P < 0.001.

Myeloid cells, particularly macrophages, play important roles in maintaining metabolic homeostasis, with the development of metabolic diseases, such as type 2 diabetes, being linked with the presence of inflammatory macrophages in the adipose tissue (28). To assess if this observed increase in *Rora* expression in myeloid cells could impact on the generation of obesity and metabolic homeostasis, adult male *Rora*^fl/fl^ and *Rora*^fl/fl^LysM^Cre/+^ mice were maintained on a HFD for 16 weeks. *Rora*^fl/fl^LysM^Cre/+^ mice had significantly (*P* < *0.05*) decreased weight gain throughout the feeding period compared to *Rora*^fl/fl^ counterparts (Figure 2A), which was associated with decreased inguinal adipose tissue weight (Figure 2B). Again, it is interesting that we specifically see a marked effect in the inguinal AT, which supports the previous expression data (Figure 1C). The reduced weight gain was associated with improved metabolic function, with significantly increased glucose tolerance (*P* < 0.001) and insulin sensitivity (*P* < 0.01), in the absence of *Rora*-expressing myeloid cells (Figure 2C). We saw no alteration in AST/ALT ratio, which serves as a marker for hepatosteatosis, suggesting that a myeloid cell deletion of *Rora* is not sufficient to convey protection against obesity-related liver damage (Figure 2D). To demonstrate that the weight loss observed is specifically due to the deletion of *Rora* in myeloid cells, and not an off-target effect of disrupting the *Rora* gene, *Rora*^fl/fl^IL-7Ra^Cre/+^ mice—a commonly used mouse to model ILC2 deficiency (29, 30)—were placed on HFD and used as an additional strain where *Rora* has been conditionally deleted in a specific cell type. In marked contrast to the reduced weight gain in mice with myeloid specific deficiency in *Rora, Rora*^fl/fl^IL-7Ra^Cre/+^ mice had increased weight gain in response to HFD (Supplementary Figures 3A,B), reinforcing cell-specific functions for *Rora* in obesity.

RORα has been studied extensively for its role in lipid metabolism. Indeed, deletion of *Rora* from the liver specifically results in exacerbated weight gain and insulin resistance associated with enhanced transcriptional activity of PPARγ resulting in uncontrolled lipogenesis (26). Furthermore, expression of genes associated with thermogenesis and fatty acid oxidation were also altered in these mice (26). However, *Rora*^sg/sg^ mice, which have a ubiquitous deletion of functional RORα, show decreased susceptibility to diet-induced obesity and an increase in thermogenesis associated with increases in UCP1 (13). We assessed the expression of *Ucp1, Elovl3, Cidea, Cptb1*, and *Ppargc1*, genes associated with thermogenesis, in both WAT and BAT deposits from obese *Rora*^fl/fl^ and *Rora*^fl/fl^LysM^Cre/+^ mice. There was significant increase in *Ucp1* and also *Elovl3* in *Rora*^fl/fl^LysM^Cre/+^ mice, suggesting that an absence of *Rora*-expressing myeloid cells is sufficient to impact on genes driving thermogenesis in the adipose tissue (Figure 2E).

### Altered Macrophage Populations in the Adipose Tissue of *Rora*^fl/fl^LysM^Cre/+^ Mice

**Figure 3:**
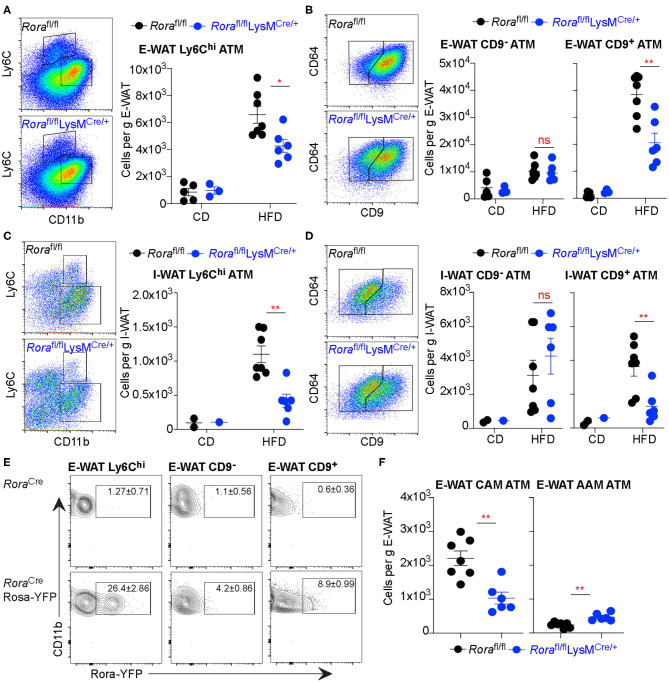
*Rora*^fl/fl^LysM^Cre/+^ mice show decreased Ly6C^hi^ ATM and fewer CD9^+^ inflammatory ATM in obese animals. Groups of *Rora*^fl/fl^ and *Rora*^*fl*/fl^LysM^Cre/+^ mice were fed a control diet (CD; *n* = 5 *Rora*^fl/fl^, *n* = 3 *Rora*^*fl*/fl^LysM^Cre/+^) or a high-fat diet (HFD; *n* = 7 *Rora*^fl/fl^, *n* = 6 *Rora*^*fl*/fl^LysM^Cre/+^) for 16 weeks and the E-WAT and I-WAT isolated and prepared for flow cytometry. Cells isolated from the E-WAT were gated as Live-dead^−^CD45^+^SiglecF^−^ and assessed for Ly6C expression **(A)**. Ly6Clo cells were further assessed for CD64 and CD9 expression **(B)**. This gating strategy was repeated in cells isolated from I-WAT (**C,D**, *n* = 2 *Rora*^fl/fl^ CD, *n* = 1 *Rora*^*fl*/fl^LysM^Cre/+^ CD; *n* = 7 *Rora*^fl/fl^ HFD, *n* = 6 *Rora*^*fl*/fl^LysM^Cre/+^ HFD). *Rora*^Cre^ and *Rora*^Cre^Rosa-YFP mice were fed a high-fat diet for 12 weeks and flow cytometry performed on E-WAT. Cells were gated as Live-dead^−^CD45^+^SiglecF^−^CD11b^+^ then gated as described previously based on Ly6C expression, then CD9 expression, as indicated, and assessed for *Rora*-YFP expression (**E**, data is representative of 3 *Rora*^Cre^ and 3 *Rora*^Cre^Rosa-YFP mice). **(F)** Cells isolated from the E-WAT from HFD fed *Rora*^fl/fl^ and *Rora*^*fl*/fl^LysM^Cre/+^ mice were stained as CAM (CD11b^+^SiglecF^−^F4/80^+^CD206^lo^) or AAM (CD11b^+^SiglecF^−^F4/80^hi^CD206^hi^; *n* = 6–7). Data is representative of mean ± SEM. Student's *t*-test: ns, not significant, *P < *0.05*, **P < *0.01*.

The immune profile of WAT largely dictates the metabolic status of the mice, ATM playing a major role. There are multiple distinct populations of macrophages within the adipose tissue, which do not fit the archetypal M1/M2 classifications (31). Recently Ly6C and CD9 expression (Ly6C^hi^ and Ly6C^lo^CD9^+^ or Ly6C^lo^CD9^−^) has been used to identify populations of ATM (31). Ly6C^hi^ macrophages are myeloid-derived adipogenic cells which reside outside the crown-like structures (CLS) in the adipose tissue, while Ly6C^lo^ macrophages reside in the CLSs that can further divided on their expression of CD9, with CD9^+ve^ cells classified as pro-inflammatory lipid laden ATM (31). We assessed the ATM populations in obese and lean *Rora*^fl/fl^ and *Rora*^fl/fl^LysM^Cre/+^ mice using Ly6C and CD9 expression to identify 3 different macrophage subtypes. While there is increase in Ly6C^hi^ ATM in mice fed a HFD in both the E-WAT and I-WAT, there were significantly fewer (*P* < 0.05 and *P* < 0.01 in E-WAT and I-WAT, respectively) Ly6C^hi^ ATM in *Rora*^fl/fl^LysM^Cre/+^ compared to *Rora*^fl/fl^ mice (Figures 3A,C). Analysis of the Ly6C^lo^ population demonstrates significantly (*P* < 0.01) decreased accumulation of CD9^+ve^ ATM in HFD fed *Rora*^fl/fl^LysM^Cre/+^ compared to *Rora*^fl/fl^ mice (Figures 3B,D), suggesting decreased lipid laden, pro-inflammatory macrophages in the AT in the absence of *Rora*-expressing myeloid cells.

*Rora*-YFP mice were used to investigate RORα expression in the 3 ATM populations. *Rora*-YFP cells were present in both the Ly6C^hi^ and CD9^+^ ATM populations (Figure 3E). The Ly6C^hi^ ATM population are myeloid derived and supports normal adipose tissue function, angiogenesis and adipogenesis (31), the relatively high expression in these cells suggests a possible role for RORα maintaining adipose tissue architecture and function. There was increased expression in the CD9^+^ population, supporting a possible role for RORα in inflammation within the context of the adipose tissue, which may be linked with its role in metabolic dysregulation. Furthermore, using archetypal classically activated macrophage (CD11b^+^SiglecF^−^F4/80^+^CD206^lo^; CAM) and alternatively activated macrophage (CD11b^+^SiglecF^−^F4/80^hi^CD206^hi^; AAM) phenotyping there were decreased CAM and increased AAM in *Rora*^fl/fl^LysM^Cre/+^ compared to *Rora*^fl/fl^ mice (Figure 3F). These data confirm the potential for important functional roles for *Rora*-expressing macrophages in the adipose tissue.

### Decreased Inflammatory Monocytes in the Blood in the Absence of RORα

**Figure 4:**
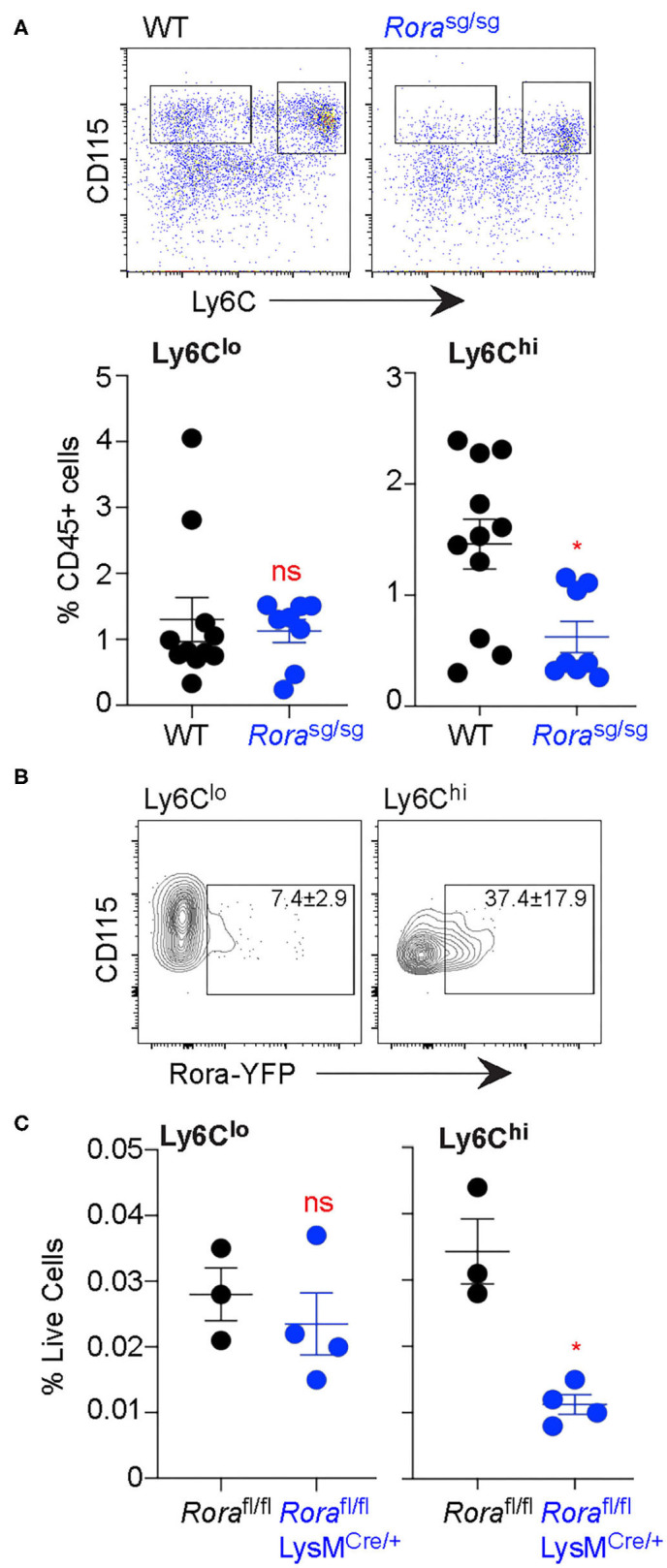
Ly6C^hi^ monocytes are decreased in the blood in the absence of myeloid cell expression of *Rora*. Blood was collected from age-matched C57Bl6/J WT and C57Bl6/J *Rora*^sg/sg^ mice by submandibular bleed. **(A)** Ly6C expression was quantified on blood CD45^+^CD115^+^ monocytes (*n* = 8 WT; *n* = 11 *Rora*^sg/sg^). **(B)** *Rora*-YFP expression was quantified on Ly6C^lo^ and Ly6C^hi^ blood CD45^+^CD115^+^ monocytes (data is representative of 3 *Rora*^Cre^Rosa-YFP mice). **(C)** Ly6C expression was quantified on blood monocytes isolated from *Rora*^fl/fl^ and *Rora*^fl/fl^LysM^Cre/+^ mice (*n* = 3 *Rora*^fl/fl^; *n* = 4 *Rora*^fl/fl^LysM^Cre/+^). Data is representative of mean ± SEM. Student's *t*-test: ns, not significant, *P < *0.05*.

While there are populations of resident ATM, the populations we assessed were myeloid-derived (28, 31). Indeed, the expression of Ly6C on ATM indicate that they are monocyte-derived and recently recruited to the adipose tissue (32). With a large proportion of Ly6C^hi^ ATM expressing *Rora*, we assessed blood monocyte populations in the absence of RORα. Using Ly6C as a marker of inflammatory blood monocytes (17), there was a significant (*P* < 0.05) reduction in Ly6C^hi^ inflammatory blood monocytes in *Rora*^sg/sg^ mice compared to WT counterparts (Figure 4A). Once again, it is the Ly6C^hi^ monocyte population that show a higher percentage of *Rora*-YFP expression, compared to Ly6C^lo^ monocytes (Figure 4B), suggesting that *Rora* expression is higher in the inflammatory monocyte population. To confirm the observed decrease in Ly6C^hi^ blood monocytes is due to *Rora* expression in myeloid cells specifically, rather than any downstream effects of a ubiquitous RORα deletion, we assessed blood monocytes in *Rora*^fl/fl^LysM^Cre/+^ mice. We see a comparable significant (*P* < 0.05) decrease in Ly6C^hi^ monocytes in *Rora*^fl/fl^LysM^Cre/+^ to that observed in *Rora*^sg/sg^ mice (Figure 4C), confirming that the decrease in this inflammatory monocyte population is due to *Rora* expression in the population.

### RORα Expression Is Higher in Myeloid-Derived Macrophages

**Figure 5:**
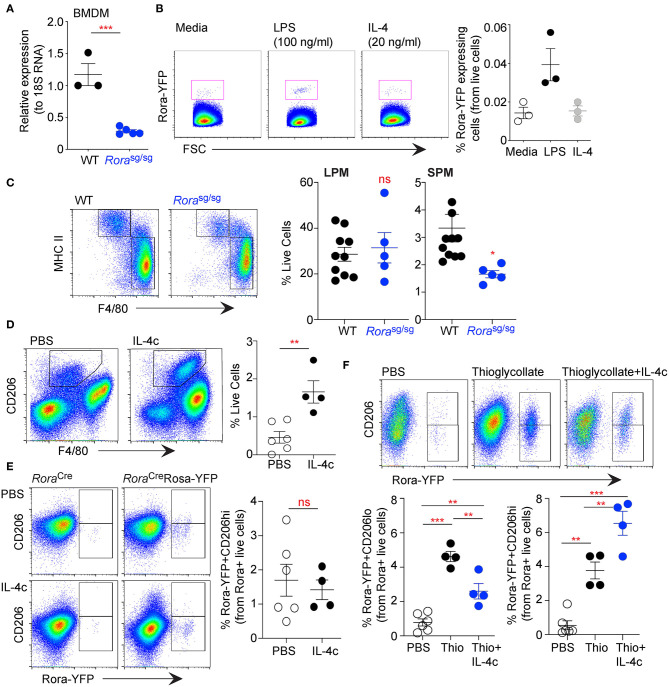
RORα acts specifically on myeloid-derived macrophages, with limited effect on tissue resident cells. Bone-marrow derived macrophages (BMDM) were isolated from age-matched WT and *Rora*^sg/sg^ mice and expression of *Rora* determined by qRT-PCR relative to 18S (**A**, *n* = 3 WT, *n* = 5 *Rora*^sg/sg^). BMDM isolated from *Rora*^Cre^Rosa-YFP mice were stimulated with media-alone, LPS (100 ng/ml) or IL-4 (20 ng/ml) for 48 h and YFP^+ve^ cells determined by flow cytometry (**B**, *n* = 3). Peritoneal exudate cells were collected from age-matched WT and *Rora*^sg/sg^ mice by peritoneal lavage and stained for flow cytometry as small peritoneal macrophages (SPM; Live/dead^−^CD45^+^CD11b^+^SiglecF^−^F4/80^+^MHC class II^hi^) or large peritoneal macrophages (LPM; Live/dead^−^CD45^+^CD11b^+^SiglecF^−^F4/80^hi^MHC class II^lo^) (**C**; *n* = 10 WT; *n* = 5 *Rora*^sg/sg^). *Rora*^Cre^Rosa-YFP mice were injected i.p. with PBS (*n* = 6) or IL-4c (*n* = 4) on days 0 and 2 **(D,E)**, or thioglycollate (day 0; *n* = 4) with or without IL-4c (days 0 and 2; *n* = 4) **(F)**. Increase in CD206^+^ macrophages in the peritoneal cavity of mice following IL-4c treatment (**D**, gated as Live/dead^−^CD45^+^CD11b^+^SiglecF^−^F4/80^+^CD206^+^). Flow cytometry was performed on LPM (gating as above; **E**) or SPM (gating as above; **F**) assessing *Rora*-YFP expression on CD206^hi^ and CD206^lo^ cells. Data is representative of mean ± SEM. Student's *t*-test or ANOVA **(E)** was used for statistical analysis: ns, not significant, *P < *0.05*, **P < *0.01*, ***P < 0.001.

Data presented thus far has demonstrated that *Rora* expressed in macrophages and monocytes are predominately of a myeloid origin. Indeed, there is decreased *Rora* gene expression in BMDM isolated from *Rora*^sg/sg^, compared to cells isolated from WT mice (Figure 5A). BMDM from *Rora*-YFP reporter mice had a small population of *Rora*-YFP^+ve^ cells, that expanded upon stimulation with LPS, but not IL-4, suggesting that classical inflammatory stimuli can drive expression of *Rora* in these cells (Figure 5B).

Studies above focused on myeloid-derived cells, to assess the potential impact of RORα on tissue resident cells we have used the peritoneal cavity as a source of macrophages. The peritoneal cavity has a resident population of macrophages, which are yolk sac derived and seeded into tissues before birth and maintained by self-renewal and local proliferation (33, 34). Assessment of peritoneal macrophage populations from *Rora*^sg/sg^ shows that while the small peritoneal macrophage (SPM; MHC class II^hi^F4/80^lo^) population is reduced in *Rora*^sg/sg^ compared to WT mice, the large peritoneal macrophage population (LPM; MHC class II^+^F4/80^hi^) is comparable between the strains (Figure 5C). This observation was also apparent in peritoneal cells in *Rora*^fl/fl^LysM^Cre/+^ when compared to *Rora*^fl/fl^ mice (data not shown). As the SPM population is monocyte-derived, whereas the LPM are the resident population, it indicates that monocyte-derived macrophages are altered in the absence of RORα with tissue-resident macrophages unaffected.

Studies herein have suggested a role for RORα in monocyte-derived inflammatory macrophages, in the periphery and in the adipose tissue. To further confirm these observations we have used a method using complexed IL-4 with IL-4R (IL-4c)-alone and in combination with thioglycollate to assess the polarization of resident macrophages as well as recruitment and polarization of monocyte derived macrophages (20). Injection of IL-4c into the peritoneal cavity resulted in a significant (*P* > 0.01) increase in CD206^+^ macrophages confirming the efficacy of IL-4c (Figure 5D). *Rora*-YFP reporter mice had modest *Rora*-YFP expression in the resident population, with *Rora* expression not altered by polarizing the cells to an AAM phenotype by the addition of IL-4c (Figure 5E). However, while the injection of thioglycollate drives the recruitment of monocyte-derived macrophages with a significant (*P* < 0.001) increase in *Rora*-YFP in the CD206^lo^ SPM population, thioglycollate and IL-4c in combination had reduced (*P* < 0.01) cells in the peritoneum (Figure 5F). While thioglycollate or thioglycollate+IL-4c injection both increased, *P* < 0.01 and *P* < 0.001, respectively vs. PBS, *Rora*-YFP^+ve^ AAM polarized monocyte in the peritoneum, there was greater (*P* < 0.01) AAM in mice received thioglycollate+IL-4c in combination (Figure 5F). This suggests that RORα is expressed in monocyte-derived macrophages in both CAM and AAM polarization conditions.

## Discussion

Studies on RORα have identified the transcription factor as vital in many aspects of neural function, cellular development, immune regulation, metabolism and circadian rhythm. Herein we demonstrate a novel role for RORα in promoting metabolic dysfunction in a mouse model of obesity, through expression in myeloid-derived inflammatory and metabolically active macrophages in the adipose tissue. Further interrogation suggests that RORα is expressed and functional predominantly in myeloid-derived monocytes, with limited expression in tissue-resident macrophages.

RORα has a known role in the progression of metabolic disease, with *Rora*^sg/sg^ mice refractory to weight gain and glucose intolerance associated with feeding a high-fat diet (3). This has been widely linked to the ability of RORα to regulate the metabolism of glucose and lipids, indeed, cholesterol is one of the ligands for RORα (3, 4, 35) as well as regulate genes associated with thermogenesis (36). Indeed, there were alterations in expression of metabolic genes in obese *Rora*^fl/fl^LysM^Cre/+^ mice when compared to *Rora*^fl/fl^ mice, particularly in genes involved in adipose tissue browning, fatty acid oxidation and thermogenesis. While BAT is thermogenic, with high levels of fatty acid oxidation and production of heat, brown adipocytes can also be found in the WAT, through induction of a thermogenic program. This is a potential target in the treatment of obesity and metabolic dysfunction, by promoting a process that favors the elimination of fatty acids rather than their storage. Indeed, studies have previously implicated RORα in the thermogenic process, with *Rora*^sg/sg^ mice expressing high levels of UCP1 in the adipose tissue suggesting that RORα inhibits the thermogenic program in WAT in a circadian manner (13).

Previous studies on the potential role for RORα in obesity and metabolic dysfunction have utilized staggerer RORα deficient mice mutant or conditional mice with ubiquitous deletion. Herein, we show that a deletion specifically in the myeloid cells drives a reduction in adiposity and improved metabolic parameters. This suggests that RORα-expressing myeloid cells are playing a vital role in these processes. Indeed, in addition to the observed effects on gene expression in the adipose tissue, we also see a modulation of the immune repertoire in the AT in the absence of RORα expressing myeloid cells. An increased percentage of Ly6C^hi^ macrophages in the adipose tissue express *Rora*-YFP, with these cells significantly decreased in the adipose tissue of obese *Rora*^fl/fl^LysM^Cre/+^ mice. We also see a decrease in CD9^+^ ATM in the absence of *Rora*-expressing myeloid cells, with these cells considered an inflammatory population (31). The reduction in the CD9^+^ population is interesting as these cells are associated with lipid storage. As there was an increase in genes associated with fatty acid oxidation in the adipose tissue of obese *Rora*^fl/fl^LysM^Cre/+^ (Figure 2D), it support that RORα may play a role suppressing these genes in the ATM as well as the adipocytes.

Resident and circulating monocytes are derived from different origins, with resident macrophages, including peritoneal resident macrophages, derived from an embryonic precursor and maintained by self-proliferation and renewal (33, 34, 37). Additionally, circulating monocytes can infiltrate the tissue, where they differentiate to an inflammatory or anti-inflammatory phenotype dependent upon the local cytokine and mediator milieu they are exposed to (38). Thus, resident and inflammatory macrophages are both developmentally and functionally distinct populations (39). Interestingly the Ly6C^hi^ population of ATM are thought to be recently recruited into the adipose tissue from the blood, retaining Ly6C expression, approximately 25% of these cells express *Rora*-YFP. Using the peritoneal cavity as a source of resident macrophages, the number and phenotype of this population is comparable in the absence of functional RORα, however the SPM, a monocyte-derived population, are significantly decreased in number. This suggests that during the embryonic stage where the resident cells are initially seeded, there is no apparent role for RORα in the generation of resident peritoneal macrophages, however, RORα does appear to impact on the recruitment of infiltrating monocytes into the peritoneal cavity. Indeed, we have previously noticed circulating levels of CCL2, a chemokine well-defined as a recruitment signal for monocytes (40), are significantly decreased in *Rora*^sg/sg^ mice. These studies suggest a potential differential role for RORα in resident and infiltrating macrophages, however, we have only addressed peritoneal macrophages in this study, other tissue macrophages would need to be explored to strengthen this hypothesis.

Our data support a multi-faceted and cell specific role for RORα in immune regulation beyond an ability to inhibit inflammatory signaling. While RORα may promote intercellular anti-inflammatory signaling pathways, its ability to shape the immune cell repertoire and promote recruitment and activation of pro-inflammatory macrophages demonstrates that RORα also promotes inflammation. These data demonstrate new functions for RORα in macrophage activation and differentiation that is relevant to the associations of *RORA* with inflammatory and metabolic disease in man.

## Data Availability Statement

The raw data supporting the conclusions of this article will be made available by the authors, without undue reservation.

## Ethics Statement

The studies involving human participants were reviewed and approved by St. Vincent's University Hospital, Dublin, Ethics committee. The patients/participants provided their written informed consent to participate in this study. The animal study was reviewed and approved by Trinity College Dublin's BioResources ethical review board.

## Author Contributions

EH and PF: conceptualization and funding acquisition. EH and JR: methodology. EH, JR, and RB: investigation. AH, DO'S, and LO'N: providing reagents. EH: writing—original draft. All authors: writing—review and editing.

## Conflict of Interest

The authors declare that the research was conducted in the absence of any commercial or financial relationships that could be construed as a potential conflict of interest.

## Supplementary Material

Supplementary Figure 1 Fluorescence-activated cell sorting confirms *Rora* expression in YFP+ve cells. **(A)** Splenocytes were prepared from *Rora*^Cre^Rosa-YFP mice and stained with CD45. The gating strategy isolates doublets and dead cells. The resultant CD45+ cells are sorted based on YFP expression, with a YFP-ve and YFP+ve population collected. The purity of the YFP+ve population is ~90%. **(B)** RNA was isolated from the sorted YFP-ve and YFP+ve populations and expression of *Rora* quantified relative to 18S. Data is representative of 3 independent sorts.

Supplementary Figure 2 Macrophages are the predominate Rora-YFP expressing cell in the adipose tissue. *Rora*^Cre^Rosa-YFP mice were fed a high-fat diet for 12 weeks and flow cytometry performed on epididymal white adipose tissue. **(A)** Cells were gated as Live/dead^−^CD45^+^*Rora*-YFP^+^ and the cells within the *Rora*-YFP gate assessed. The percentage of CD4+ T cells (Rora-YFP^+^CD45^+^CD4^+^), ILC2(*Rora*-YFP^+^CD45^+^CD4^−^Lineage^−^Sca-1^+^IL-7Ra^+^), eosinophils (*Rora*-YFP^+^CD45^+^CD4^−^CD11b^+^SiglecF^+^), and macrophages (*Rora*-YFP^+^CD45^+^CD4^−^CD11b^+^SiglecF^−^F4/80^+^) is expressed as a proportion of *Rora*-YFP^+^CD45^+^) cells. **(B)** *Rora*-YFP expression was assessed in Live/dead^−^CD45^−^ cells in the stromal vascular fraction of the E-WAT of *Rora*^Cre^Rosa-YFP reporter mice and control *Rora*^Cre^ mice. Data is representative of 3 mice.

Supplementary Figure 3 *Rora*^fl/fl^IL-7RaCre mice display exacerbated weight gain when fed a high-fat diet. Groups of *Rora*^fl/fl^ and *Rora*^*fl*/fl^IL-7Ra^Cre/+^ mice were fed a high-fat diet (HFD; 60% fat) for 14 weeks and weight monitored weekly, percentage weight gain was calculated from the starting weight of each animal **(A)**. E-WAT weight was determined after 14 weeks on HFD **(B)**. All data is representative of mean ± SEM (*n* = 3 *Rora*^fl/fl^; *n* = 7 *Rora*^*fl*/fl^IL-7Ra^Cre/+^ mice). Student's *t*-test: ^*^*P* < *0.05*.
