# Supplementary figures and images for: Role for Retinoic Acid-Related Orphan Receptor Alpha (RORα) Expressing Macrophages in Diet-Induced Obesity
Source: Front Immunol. 2020 Aug 27;11:1966. doi: 10.3389/fimmu.2020.01966 (PMC7482427; doi:10.3389/fimmu.2020.01966)

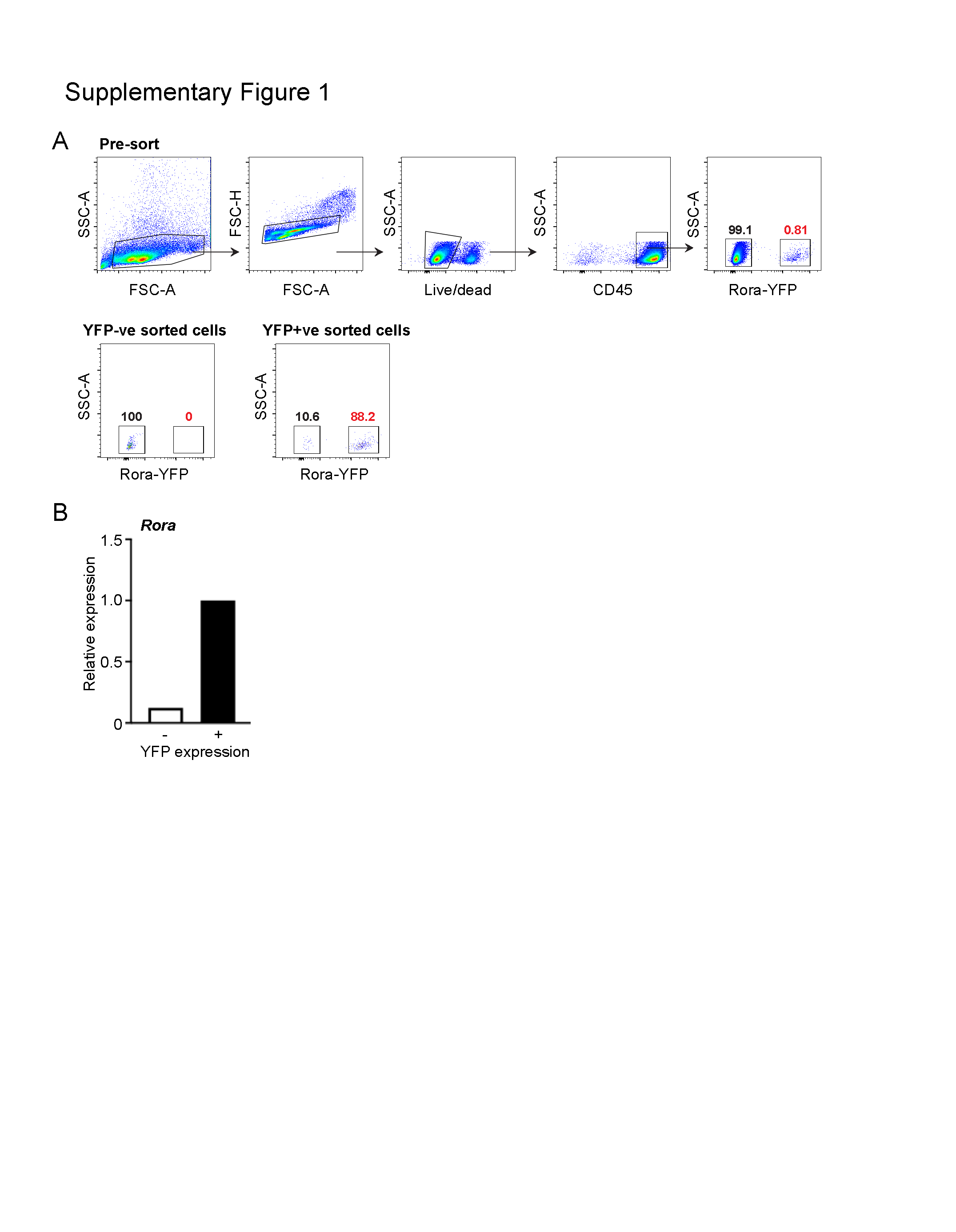

Supplement: Supplementary Figure 1 — Fluorescence-activated cell sorting confirms Rora expression in YFP+ve cells. (A) Splenocytes were prepared from RoraCreRosa-YFP mice and stained with CD45. The gating strategy isolates doublets and dead cells. The resultant CD45+ cells are sorted based on YFP expression, with a YFP-ve and YFP+ve population collected. The purity of the YFP+ve population is ~90%. (B) RNA was isolated from the sorted YFP-ve and YFP+ve populations and expression of Rora quantified relative to 18S. Data is representative of 3 independent sorts. [file Image_1.tiff]

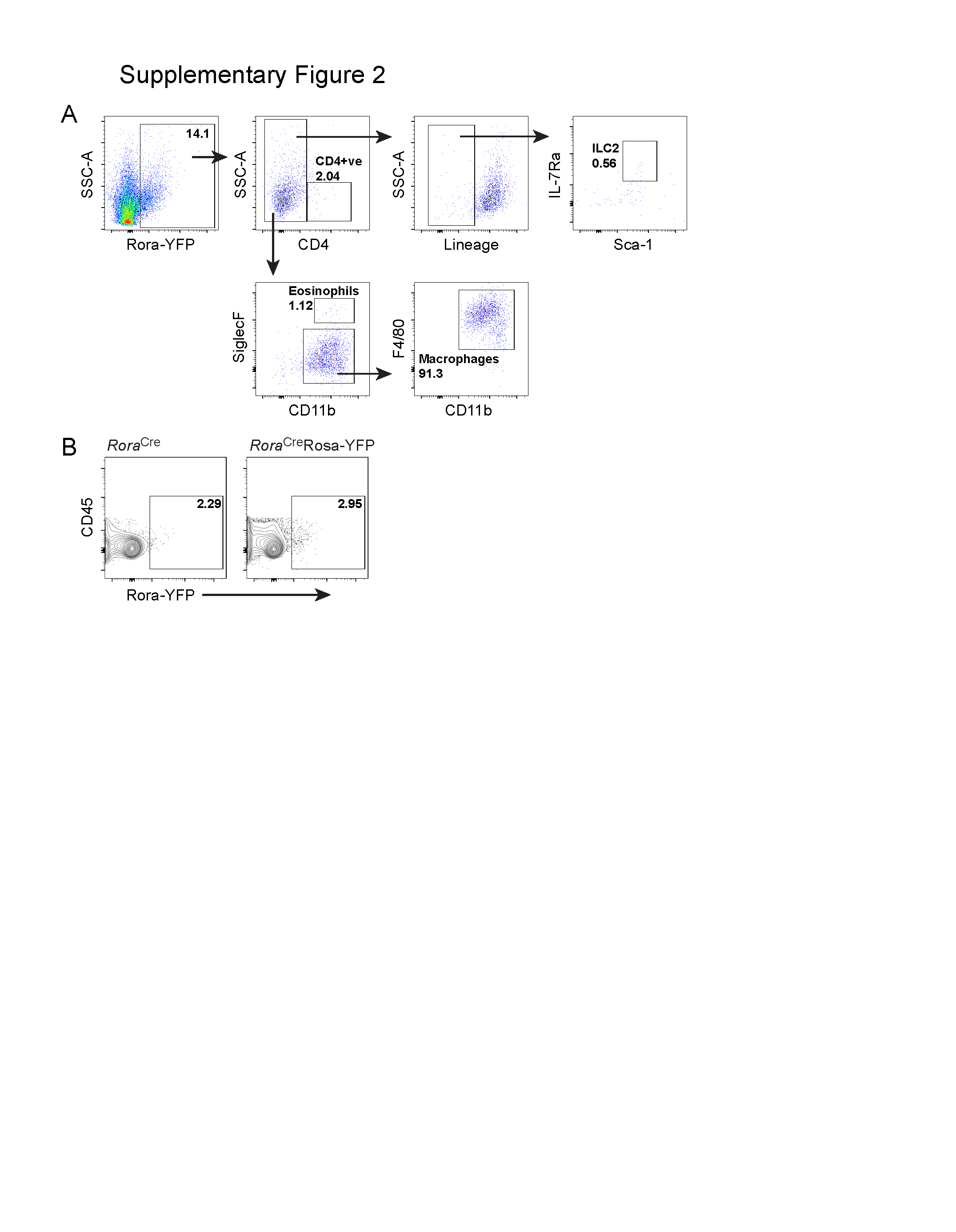

Supplement: Supplementary Figure 2 — Macrophages are the predominate Rora-YFP expressing cell in the adipose tissue. RoraCreRosa-YFP mice were fed a high-fat diet for 12 weeks and flow cytometry performed on epididymal white adipose tissue. (A) Cells were gated as Live/dead−CD45+Rora-YFP+ and the cells within the Rora-YFP gate assessed. The percentage of CD4+ T cells (Rora-YFP+CD45+CD4+), ILC2(Rora-YFP+CD45+CD4−Lineage−Sca-1+IL-7Ra+), eosinophils (Rora-YFP+CD45+CD4−CD11b+SiglecF+), and macrophages (Rora-YFP+CD45+CD4−CD11b+SiglecF−F4/80+) is expressed as a proportion of Rora-YFP+CD45+) cells. (B) Rora-YFP expression was assessed in Live/dead−CD45− cells in the stromal vascular fraction of the E-WAT of RoraCreRosa-YFP reporter mice and control RoraCre mice. Data is representative of 3 mice. [file Image_2.TIFF]

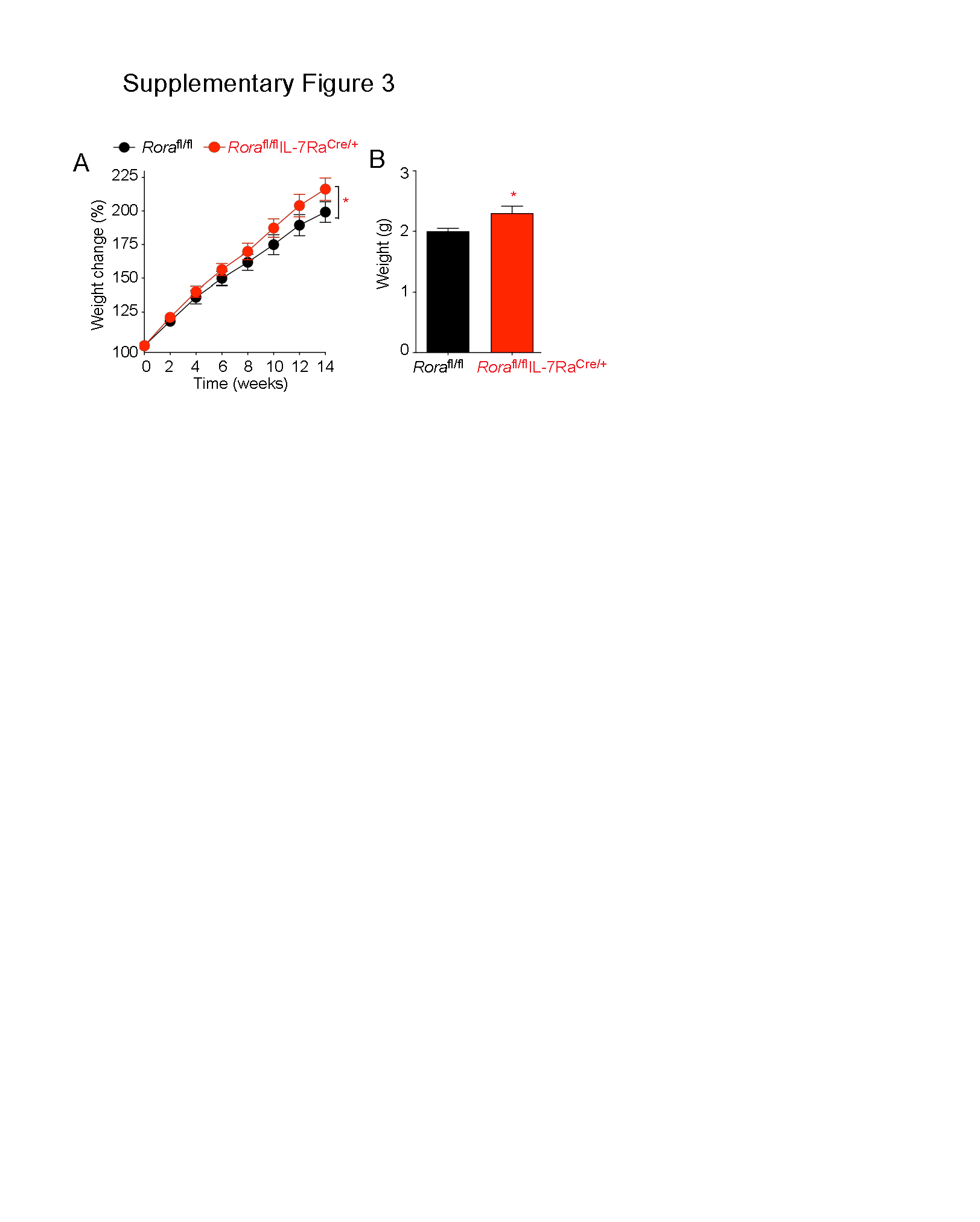

Supplement: Supplementary Figure 3 — Rorafl/flIL-7RaCre mice display exacerbated weight gain when fed a high-fat diet. Groups of Rorafl/fl and Rorafl/flIL-7RaCre/+ mice were fed a high-fat diet (HFD; 60% fat) for 14 weeks and weight monitored weekly, percentage weight gain was calculated from the starting weight of each animal (A). E-WAT weight was determined after 14 weeks on HFD (B). All data is representative of mean ± SEM (n = 3 Rorafl/fl; n = 7 Rorafl/flIL-7RaCre/+ mice). Student's t-test: *P < 0.05. [file Image_3.tiff]
